# Blockchain Based Authentication and Cluster Head Selection Using DDR-LEACH in Internet of Sensor Things

**DOI:** 10.3390/s22051972

**Published:** 2022-03-02

**Authors:** Sana Amjad, Shahid Abbas, Zain Abubaker, Mohammed H. Alsharif, Abu Jahid, Nadeem Javaid

**Affiliations:** 1 Department of Computer Science, COMSATS University Islamabad, Islamabad 44000, Pakistan; sanaamjad702@gmail.com (S.A.); shahidabbas1260@gmail.com (S.A.); zainmalik.gcuf@gmail.com (Z.A.); 2 Department of Electrical Engineering, Sejong University, Seoul 05006, Korea; malsharif@sejong.ac.kr; 3 School of Electrical Engineering and Computer Science, University of Ottawa, Ottawa, ON K1N 6N5, Canada; ajahi011@uottawa.ca; 4 School of Computer Science, University of Technology Sydney, Ultimo, NSW 2007, Australia

**Keywords:** blockchain, clustering, authentication, malicious node detection, LEACH protocol, service provisioning, interplanetary file system, security

## Abstract

This paper proposes a blockchain-based node authentication model for the Internet of sensor things (IoST). The nodes in the network are authenticated based on their credentials to make the network free from malicious nodes. In IoST, sensor nodes gather the information from the environment and send it to the cluster heads (CHs) for additional processing. CHs aggregate the sensed information. Therefore, their energy rapidly depletes due to extra workload. To solve this issue, we proposed distance, degree, and residual energy-based low-energy adaptive clustering hierarchy (DDR-LEACH) protocol. DDR-LEACH is used to replace CHs with the ordinary nodes based on maximum residual energy, degree, and minimum distance from BS. Furthermore, storing a huge amount of data in the blockchain is very costly. To tackle this issue, an external data storage, named as interplanetary file system (IPFS), is used. Furthermore, for ensuring data security in IPFS, AES 128-bit is used, which performs better than the existing encryption schemes. Moreover, a huge computational cost is required using a proof of work consensus mechanism to validate transactions. To solve this issue, proof of authority (PoA) consensus mechanism is used in the proposed model. The simulation results are carried out, which show the efficiency and effectiveness of the proposed system model. The DDR-LEACH is compared with LEACH and the simulation results show that DDR-LEACH outperforms LEACH in terms of energy consumption, throughput, and improvement in network lifetime with CH selection mechanism. Moreover, transaction cost is computed, which is reduced by PoA during data storage on IPFS and service provisioning. Furthermore, the time is calculated in the comparison of AES 128-bit scheme with existing scheme. The formal security analysis is performed to check the effectiveness of smart contract against attacks. Additionally, two different attacks, MITM and Sybil, are induced in our system to show our system model’s resilience against cyber attacks.

## 1. Introduction

The wireless sensors networks (WSNs) play an important part in the Internet of sensors things (IoST) [1]. IoST is useful in sensing data from the environment and is used in the field of energy trading, surveillance, smart grids, etc., [2,3]. It connects with the Internet and automates the monitoring system without any involvement from a third party. The IoST network consists of sensor nodes that perform environmental monitoring [4]. However, the sensor nodes in the WSNs face the issue of non-repudiation, limited resources, presence of malicious nodes, etc., [5,6,7]. Many studies are proposed to solve these aforementioned issues [8,9,10]. However, these studies have issues of single point of failure (SPOF) and performance bottlenecks due to their centralized architecture.

To overcome these aforementioned issues, many researchers provide different mechanisms to remove third parties by introducing blockchain in the WSNs. Blockchain is a secure and decentralized protocol that solves many issues such as SPOF, a third party involvement, etc., [11]. Moreover, the distributed and tamper-proof ledger in blockchain solves trust issues between unknown entities. The transactions that are performed by entities in the network are confirmed by the miners. These transactions are validated by the miners using various consensus mechanisms, such as proof of work (PoW) [12], proof of authority (PoA), proof of stake, etc., [13]. In the PoW mechanism, all the nodes participate in solving the mathematical puzzle. The node that solves it first validates the transactions. The blockchain is created by validating and storing the transactions. Moreover, a smart contract is used in blockchain in which all the terms and conditions are finalized. Additionally, it eliminates the third party. Moreover, blockchain provides security in the network by malicious nodes’ detection through Merkle tree [14,15] and also through different techniques, such as trust evaluation of nodes, etc., [16,17,18].

Many blockchain-based schemes are proposed to solve the issues of single point of failure, huge monetary cost, and performance bottlenecks [19,20,21,22]. However, the data of all these networks are stored on blockchain, which is very costly. When 1MB of data are stored on blockchain, it costs USD 14151.68  [23]. Moreover, PoW consensus algorithm is used in [21,22], which is not suitable for resource constrained environment.

In IoST networks, routing is an important aspect in which nodes communicate and transmit data from source to the destination. The transmitted data are controlled by different nodes in IoST that are sensor nodes, CHs and base stations (BSs). In [19], the data are processed by CHs and are forwarded to the BSs. However, authentication of network nodes is not performed. Therefore, any node can enter the network and behave maliciously. Moreover, in [19], no cost-effective data storage mechanism is proposed, which leads to expensive data storage in blockchain. As data are permanently stored on blockchain then the issues of limited storage arises. Additionally, in an IoST network, CHs fail due to high energy depletion, which affects the whole network’s performance. In [21], no mechanism is proposed for the selection of new CHs. Furthermore, in PoW, the miners solve the puzzle for validating the transactions and adding the blocks into the blockchain [17,22]. This mining process takes considerable time due to puzzle’s complexity, which ultimately increases network’s computational cost. This paper is the extension of [24] and the contributions in the proposed work are as follows:

- The identity authentication of nodes is performed to remove the external unauthenticated nodes;
- CHs are selected from ordinary nodes using the proposed minimum distance, highest degree, and highest residual energy (DDR) based LEACH protocol;
- IPFS is used to provide distributed storage for IoST;
- A payment method is proposed to motivate IPFS for long term data storage;
- A blockchain based secure service provisioning mechanism is proposed;
- An advanced symmetric encryption algorithm (AES) 128-bit is used for the integrity of data;
- Comparison of DDR-LEACH is performed with the LEACH protocol;
- Formal security analysis is performed for the smart contract to check its effectiveness;
- Man in the middle (MITM) and Sybil attacks are induced in the network, which show that our proposed system is resilient against these attacks.

The rest of the paper is organized as follows. The related work is discussed in Section 2 and Table 1. The proposed system model is presented in Section 3. The simulation results and the validation of system model are discussed in Section 4. The formal security analysis is discussed in Section 5. Section 6 contains the conclusion of the proposed work and future work.

## 2. Related Work

The studies related to the blockchain integrated with WSNs are discussed in this section. The studies are categorized based on the limitations they have addressed.

### 2.1. Nodes’ Authentication

The sensor nodes perform an important role in performing many tasks in IoT networks and nodes’ identities authentication is one of them. The nodes in the IoT network work together and provide the services to the buyers. However, the nodes’ identities authentication is not performed in [13,21], which leads to malicious nodes becoming the part of the network and affecting its performance.

Authentication is required to restrict unauthenticated nodes from entering the network. The unauthenticated nodes behave maliciously by tampering data during routing, as well as refusing to forward data packets toward the destination. Therefore, in [20], the authors propose a lightweight authentication mechanism for WSNs. The sensor nodes use unique sequence numbers during data transmission based on the concept of a Merkle tree. Secure hashing algorithm 1 (SHA-1) is used to authenticate the nodes. Although in [25], the network nodes are authenticated using routing protocol. However, due to centralized authority, a trust issue is created. In [26], the wireless body area network is comprised of sensor nodes, which collect the information of human body parts and publicly forward it to the local node. Health is a very critical and sensitive matter, and the malicious nodes can enter in the network and misuse the data. However, these nodes are not authenticated in the network.

### 2.2. Lack of Data Storage

The authors in [27] propose an incentive based data storage mechanism. Each node stores data in the blockchain; however, computational cost increases due to the usage of PoW consensus mechanism. Although, in [29], mining is performed using PoW. The PoW increases the computational cost. Therefore, a lightweight blockchain network is proposed to reduce blockchain storage and computational requirements in IoST environment. The blockchain is merged with IoTs in [30] to aggregate the blocks’ header information and transmit it to the IoT nodes. However, keeping a copy of data in a resource constrained network is not appropriate.

### 2.3. Lack of Data Privacy

In [19], no technique is proposed to prevent the network data from being stolen by malicious nodes. Additionally, in [31], the products are controlled and monitored by workers in the industry. However, the issue of data transparency is created. The important information of the products may be stolen by the workers. In addition, the misuse of important products’ record is also possible. Additionally, in the WSNs, the security of data and its privacy is compromised [13]. In [32], the authors state that collecting the information from crowdsensing is essential for the network nodes. However, privacy protection of the data is not considered.

Whereas, in [33], the data-driven network is converged with WSN and the reserving information is copied for sharing it in the network. However, the security of data is being compromised by the malicious nodes. Moreover, in [22], the growth of IoT in the smart city creates data latency, scalability, and huge bandwidth issues. Therefore, hybrid blockchain network is proposed and SDN controllers are used as an interface between the IoT. Additionally, digital signatures are used for the data security in the network. The sensor nodes transmit the data to IoT nodes in [37]; however, no mechanism for data security is proposed, which causes data security issue.

### 2.4. Lack of Resources

In [27], the chances of malicious nodes’ existence are high in the network, which do not allow the legitimate nodes to participate in the network. Additionally, blockchain technology is used for different purposes, such as content caching in [28]. Moreover, in [27,28], the authors use PoW consensus mechanism, in which high computational power is required. The blockchain is integrated with IoT for secure routing in [30]. However, the nodes in the network have very low storage capability and these resource constrained nodes cannot keep the copied records in them. Whereas, in [38], PoW is replaced with Tangle based technology to provide fast and secure information. However, the frequency of transaction is very low. Moreover, the IoT sensor nodes’ energy depletes very fast due to the high computational overhead. Low computational power of IoT nodes hinders the validation of transactions.

### 2.5. Malicious Nodes’ Existence

Obtaining the exact location of sensor nodes in the network is an emerging domain nowadays. However, malicious behavior of sensor nodes lead to the broadcast of wrong location information. Due to this, the security of the network is compromised [34]. Additionally, in [17], no mechanism is proposed to detect the malicious nodes in the network. Different fields, such as manufacturing products and healthcare use IoT [35]. However, still some challenges are faced by provisioning process, such as provision of malicious services. Furthermore, the client can behave maliciously by repudiating on behalf of services. In [36], the sensor nodes find the shortest path for communication. However, there is no mechanism to secure the data and to find the malicious nodes.

### 2.6. Single Point of Failure Issue

The network performance is affected when the identity authentication of nodes is compromised. In [21], the nodes’ identity depends on central authority servers that become the reason for SPOF. Whereas, in [39], the authors integrate the software-defined networking and blockchain to detect attacks without any involvement of a third party. The data are sent directly to the centralized cloud. However, bandwidth and latency issues arise. The smart contracts in the blockchain system share the trained classifiers with the cloud layer for fusion. However, SPOF issue arises due to central authority. In [25], a centralized authority is used to authenticate the routing nodes. Due to the central authority used in the system, SPOF issue arises. In [40], the data are stored in the network by a centralized system, which leads to SPOF issue.

Table 2 presents problems identified in existing literature, their proposed solutions and their validations. An authentication scheme is proposed to prevent from the unauthenticated nodes, so that only authenticated nodes are allowed to perform an action. In DDR-LEACH protocol, motivated from [41], the highest degree node is selected, which solves the node battery issue. Moreover, IPFS is used to solve the costly data storage issue of the blockchain. IPFS stores the data cost-effectively and distributively. Additionally, the issue of high computational cost is resolved using PoA consensus mechanism.

## 3. Proposed System Model

In this section, the assumptions, components and work flow of the proposed system model are discussed.

### 3.1. Assumptions

The system model is based on following assumptions:

- BSs are considered legitimate. As they are peers of blockchain; therefore, they provide secure services to buyers;
- Symmetric keys are exchanged securely in the network.

### 3.2. System Components

**Figure 1:**
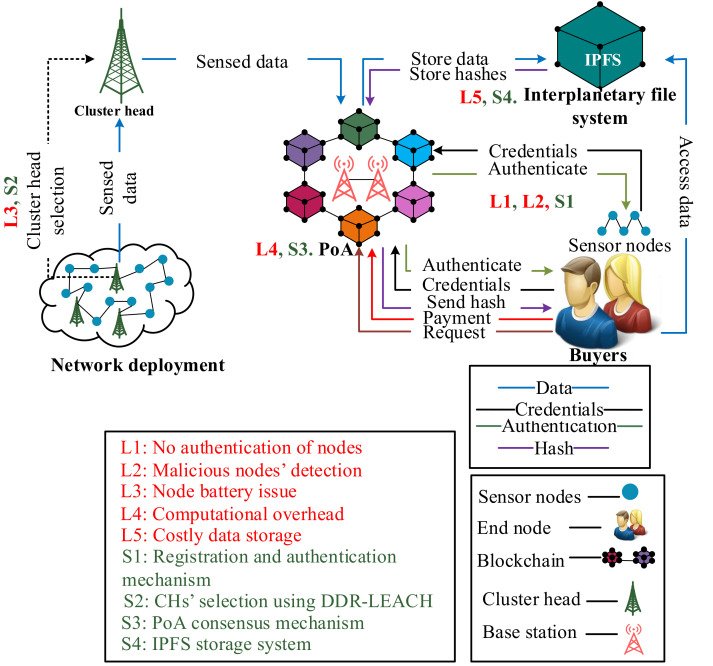
Blockchain based nodes’ authentication and CHs’ selection in IoST.

In this section, the components of the proposed system model are discussed as depicted in Figure 1. The components include IoST, buyers, IPFS, and blockchain.

*Internet of sensor things:* The IoST is an emerging technology, which consists of sensor nodes deployed for collecting the environmental data [32]. The sensor nodes sense the surrounding information such as the data of humidity, pressure, and temperature, etc., [42]. In the proposed system model, the IoST consists of sensor nodes, CHs, and BSs. Their working is described in Section 3.3.

*Buyers:* To prevent the network from malicious activities, the buyes are registered and authenticated in the blockchain network. For that purpose, registration and authentication schemes are used, motivated from [21].

*Interplanetary file system:* It is a distributed platform where data are stored in the form of chunks. Whenever the data are stored on IPFS, a hash is generated. IPFS generates the 32-bit hash in result of data storage, which is stored on the blockchain as a record.

### 3.3. Workflow of the System Model

The system model is discussed in the steps given below.

*Step 1. Initialization*: The blockchain technology introduces a smart contract, which is a digital agreement that works without the involvement of any third party. It is deployed on BSs that handle the network transactions. The blockchain is used for registering sensor nodes in the network by storing their credentials for authentication. Credentials are sent in the form of a message shown in following equation.

(1) $${\left(ID_{Node},MACAddr_{Node},Reputation_{Node}\right)}_{Packet}=Message$$

In the registration and authentication process, MAC address, ID, and Reputation of nodes are used as credentials. $Reputation_{Node}$ is the reputation value given to a specific node on the basis of its previous history of interaction with the network. If the node provides accurate data to the network, its reputation increases; otherwise, it decreases. The credentials are stored in the blockchain using a asymmetric scheme. The blockchain also keeps their addresses to prevent the network free from malicious activities. Therefore, during authentication process, the credentials are matched with already stored data. If the credentials are not matched with already stored data, then the node is considered as a malicious node.

*Step 2. CH selection*: The IoST network consists of sensor nodes, CHs, and BSs. The sensor nodes collect the data from the environment and send it to CHs for performing computation. CHs receive data from sensor nodes, process the data, and send it to BSs. BS requests IPFS for storing data; IPFS calculates hash value of the data and sends it to BS. In processing and storing the data, the energy of CHs depletes rapidly. To solve this issue, our proposed model provides a mechanism that selects CHs from ordinary nodes using DDR-LEACH. The CHs are selected based on three parameters: residual energy, minimum distance from BSs, and maximum degree of a node. If a node satisfies the criteria mentioned earlier, then it is selected as CH. CH aggregates the data packet and transmits it to the destination, as given in Algorithm 1. If more than one nodes meet the criteria, then CH is randomly selected.
**Algorithm 1:**CH selection.
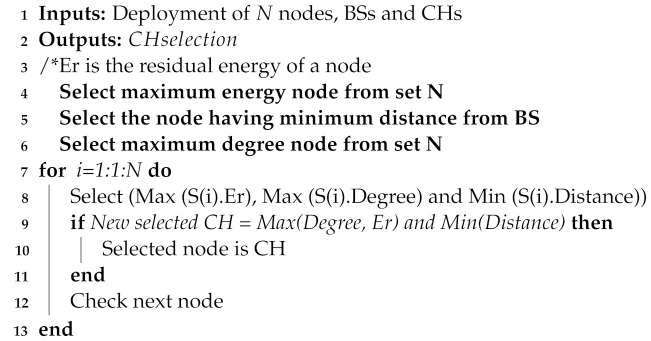


*Step 3. Nodes’ authentication*: In this step, sensor nodes are authenticated in the blockchain. The sensor nodes’ authentication is important to revoke malicious nodes from the network and provide the secure services. The messages are exchanged between the WSN nodes and the distributed ledger, i.e., blockchain, after being encrypted using asymmetric encryption. The encryption is performed over the registration data for providing more security. The node encrypts the data with the public key of the BS (known to everyone in the network) and then BS decrypts the encrypted data with its private key (known to BS only) and stores data in the blockchain. Moreover, the solution for providing the keys for encryption and decryption remains the same because the keys can neither be invalidated nor can be changed. Additionally, whenever any node performs any activity in the network, its credentials are verified by the BS. Therefore, this solution is sufficient to provide the security to the overall network. There are many papers that are based on nodes’ authentication, i.e., [43,44]. As in [43], the nodes’ identity authentication is performed using an encryption scheme. However, due to centralized authority, the SPOF and performance bottleneck issues arise. Although, in [44], a lightweight authentication scheme is used. However, due to centralized authority, the SPOF issue occurs. Few authentication-based papers are mentioned in the related work, i.e., [20,21]. As in [20], authentication of nodes is performed by their acknowledgment to the sink node. The nodes acknowledge the sink node based on their provided sequence numbers. However, during nodes’ authentication, the PoW consensus mechanism is used, which incurs high computational cost. Although, in [21], hybrid blockchain-based nodes’ authentication is performed. However, PoA is used, which incurs high computational cost. Moreover, data storage in blockchain is very costly. In our proposed system model, we have used nodes’ identity authentication using a PoA consensus mechanism, which reduces computational cost. We do not claim that this authentication model is the novel work. We have embedded this authentication model with an efficient CH selection and secure storage of sensors’ data. This integration of authentication model is a novel combination, as authentication mechanism is used in different scenarios [20,21,44]. Moreover, a distributed IPFS data storage mechanism is used to reduce overall monetary cost of the network. The authentication is performed by matching the credentials of nodes that are already stored in the blockchain.

When a node is taken over by a malicious node, then malicious node can transmit these data in the network to perform malicious activities. As we know, all legitimate nodes are registered with BSs and their credentials are already stored in the blockchain. When any malicious node transmits the data, it can be easily detected in authentication process because its credentials are not stored in the blockchain. In this way, the malicious node cannot perform malicious activities in our network by taking over any node. Moreover, when a malicious node is removed from the network, it is revoked from transmitting any kind of data in the network. The malicious node is removed and revoked by BSs because blockchain is deployed on them and they are responsible for validating the transactions. In the proposed model, PoA is used as the consensus mechanism for mining the transactions. It incurs less computational cost as compared to the traditional PoW consensus mechanism. Additionally, the miners are pre-selected nodes in PoA. The registered nodes are authenticated after the authentication request. The request contains $ID_{Node}$, $MACAddr_{Node}$, and $Reputation_{Node}$, which are already stored on the blockchain. Blockchain checks whether the credentials provided by nodes are matched with the credentials already stored or not in the blockchain. If the credentials match the provided information, the nodes become authentic and are broadcasted as legitimate nodes. Otherwise, they are broadcasted as malicious or unauthentic nodes. The step-wise process is according to Algorithm 2.

*Step 4. Data storage*: Storing the large amount of data on the blockchain is not suitable because the storage cost is high on the blockchain. The cost of storing 1 MB data on the blockchain is approximately USD 14151.68 [23]. Therefore, BSs send data to IPFS and keep its hash values and the credential of registered nodes in the blockchain. In the blockchain, the transaction of each entity is stored in a ledger. This ledger is distributed to all entities of the network. All entities act as a foundation of the network. When any transaction is performed, the ledgers of all entities are updated simultaneously. When malicious node tries to manipulate the transaction record in a ledger of any entity, then it can easily be detected because this ledger is not matched with already stored ledger. The data are stored on IPFS in the form of chunks. IPFS does not store the data for long time. Therefore, we propose a payment method for long time data storage of IPFS. The IPFS is incentivized in order to motivate the peer nodes for storing the data. The hashes are stored on the blockchain. Only authenticated nodes obtain data using the provided hash. However, the data storage on IPFS is temporary. Therefore, we propose an incentive method for IPFS to store a huge amount of data for a long time.
**Algorithm 2:**Nodes’ authentication process.
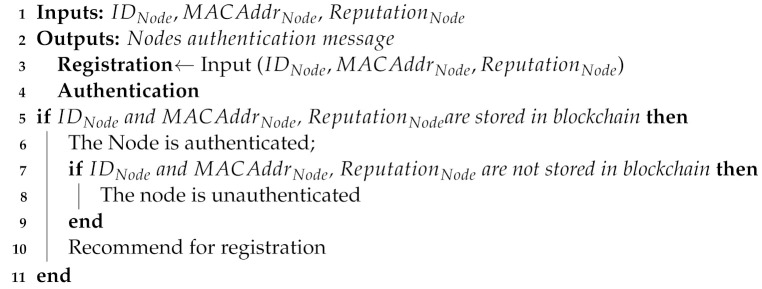


*Step 5. Service provisioning*: We use a private blockchain, deployed on BSs, to register and authenticate the nodes. In the beginning, the blockchain receives the registration request from the buyers. Then, the nodes are checked by the blockchain whether they are already registered or not. If a buyer is already registered, it discards the request. Otherwise, it allows the node for registration. Algorithm 2 shows how the authentication process works.

The already stored credentials in the blockchain are used to authenticate the buyers. A buyer must first provide its credentials for verification in the blockchain. The blockchain checks the credentials to confirm whether the node’s credentials exist in the blockchain or not. If the provided credentials match with the stored one, it is considered as an authentic user; otherwise, it is considered a malicious node, which is immediately removed from the network. Afterward, if the buyer is authenticated, then the ethers are checked according to the threshold. If ethers are enough to buy the data, the hash of the requested data is sent to the buyer; otherwise, request will be rejected. After the authentication process, the buyer receives services from the network. Whenever a buyer requests the service, BS encrypts the service with buyer’s secret key and sends the cypher text to the buyer.

**Figure 2:**
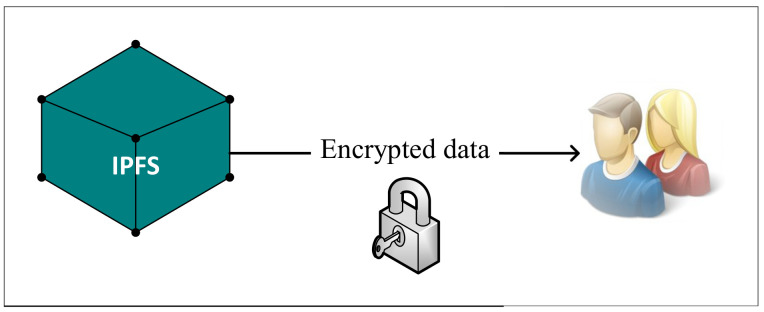
Interaction of buyers with IPFS.

The buyer receives the encrypted data and then decrypts it with the private Figure 2.

We use AES 128-bit encryption for ensuring data security in the network. Moreover, SHA-256 is used with AES encryption to ensure data integrity. Initially, the BS calculates the hash by the SHA-256 hashing algorithm and then uploads this hash on the blockchain. After this, the BS encrypts the data with a secret key and sends these encrypted data to a client. The buyer receives the data and decrypts it with the secret key provided by the sender. After decryption of data, the buyer calculates the hash by itself using SHA-256 algorithm and compares this hash with the hash already stored in blockchain. In this way, the SHA-256 hashing technique together with the AES encryption technique ensures data security and data integrity. Moreover, we have used the AES 128-bit encryption technique in our scenario because our primary goal is to provide real time data to buyers and AES 128-bit encryption consumes a very small amount of time in encryption and decryption of data. Furthermore, the efficiency of the AES 128-bit encryption scheme in terms of time is shown in Figure 10 in the simulation section.

In the proposed system model, 5% of the buying amount is given to IPFS as an incentive. The service provisioning mechanism is shown in Algorithm 3.
**Algorithm 3:** Service provisioning.
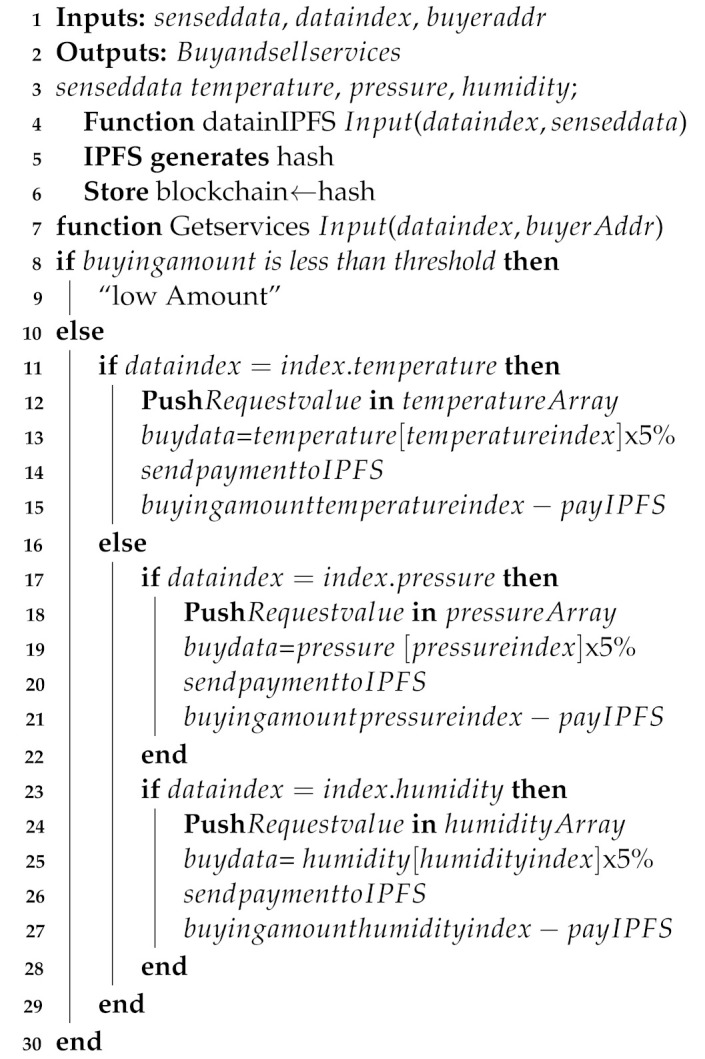


In Table 2, the mapping between limitations, proposed solutions and their validations is provided. The limitations L1 and L2 represent the unauthenticated and the malicious activities of nodes in the network, which are solved by S1. The parameter that is used to validate the nodes’ authentication is message size, which is evaluated by transaction cost. The message size indicates that how many bytes it takes to authenticate a node while the transaction cost means the cost required for nodes’ authentication. The third limitation L3 is the inefficient energy consumption of CHs. In S2, CHs are selected based on nodes’ residual energy, minimum distance from BS and degree. If these conditions are satisfied for a node, then it becomes a CH. The network lifetime, packet delivery ratio and energy consumption are used as validation parameters. L4 indicates the high computational cost when using PoW consensus mechanism. In S3, PoA consensus mechanism is used to solve this issue, which consumes less computational resources as compared to PoW. The cost incurred when using PoA is depicted by the average transaction cost. The limitation L5 shows that the data storage on the blockchain is very costly. Therefore, a large amount of data are stored in the IPFS in S4. The average transaction cost and encryption time are taken as validation parameters. The average transaction cost shows the cost used to store data on IPFS. At the same time, encryption time shows the time, which is used in encryption and decryption of data during service provisioning.

## 4. Simulation Results and Discussion

In this section, the performance evaluation of the proposed model is described. The specifications for the simulation setup include an Intel(R) Core (TM) i5-2520M CPU @ 2.50 GHz processor, 64-bit operating system, x64-based processor, and 8 GB RAM. Remix IDE is used to develop a smart contract, whereas, Ganache is used to manage the transactions. Although MetaMask is used for providing virtual currency to perform transactions. Moreover, for simulating our WSN, MATLAB is used. The use of a cryptographic scheme is simulated in Visual Studio using Python.

In the network, three types of nodes are considered for the simulations: 100 sensor nodes, 4 CHs, and 2 BSs in the network area of 100 × 100 m^2^. Moreover, there are three input data in our system model: sensed data, data index, and buyer’s address. The data are sensed by sensor nodes in the network. They send the sensed data to CHs for processing. The CHs after processing the data send it to BSs for further processing. The BS stores the data with data index. The sensed data with data index are provided by BSs. Furthermore, the buyer’s address is the Ethereum address that is provided to each buyer when it joins the network. Initially, the nodes are registered and authenticated to provide malicious nodes free network. The simulation parameters are mentioned in Table 3.

**Figure 3:**
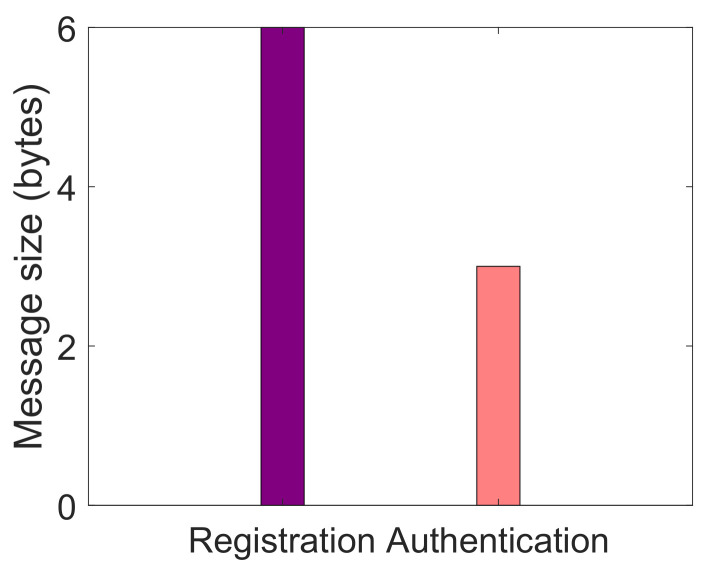
Message size.

Figure 3 illustrates the message size of network nodes. During the registration phase, unknown nodes request to become a part of the network. For the registration process, a new node first registered in the blockchain network before its participation. The nodes send their credentials to the blockchain. BS stores its credentials, and perform authentication process. The message size is large during the registration phase as compared to authentication phase because in registration phases, more resources are required to store the data on the blockchain. Whereas, in authentication phase, BS only matches the nodes’ credentials with the already stored credentials, which incurs less resources.

**Figure 4:**
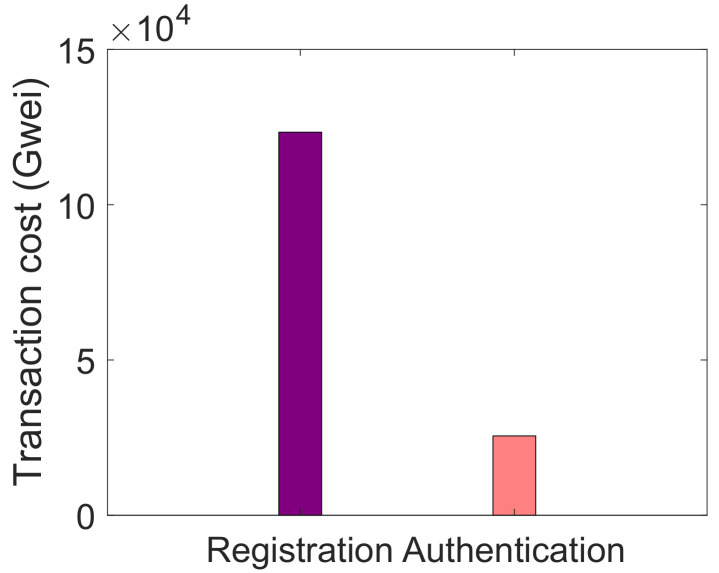
Transaction cost during registration and authentication of nodes.

Figure 4 shows the transaction cost incurred during registration and authentication phases. When smart contracts are deployed, then transaction cost is incurred. The results show that the transaction cost increases with the increase in the number of packets. Similar behavior is observed for authentication and registration phases in Figure 4 as that of Figure 3. The transaction cost increases with the increased number of nodes. The authentication process takes less transaction cost because already stored registration information has to be verified.

**Figure 5:**
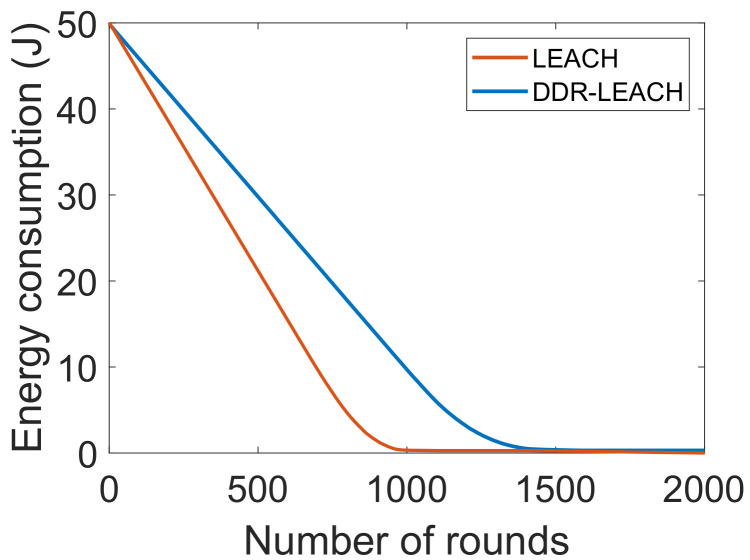
Energy consumption.

Figure 5 shows the energy consumption against the number of rounds. The comparison of LEACH protocol with DDR-LEACH is performed in terms of energy consumption. In the DDR-LEACH, energy is consumed by the nodes till 1400 rounds. Whereas, in the LEACH, the energy is consumed by the nodes till 1000 rounds. The energy consumption is high in LEACH because random CHs’ selection is performed. The sensor nodes near to the BS may not participate in the network and may not be selected as CHs. Therefore, maximum energy is utilized by any of the nodes to send the data packets from source to destination. As compared to LEACH, the maximum energy is consumed by the nodes till 1400 rounds. The DDR-LEACH considers the three parameters that are maximum degree, minimum distance, and minimum energy consumption to select the CHs. The energy usage is efficient in the starting rounds because CHs are alive and working. CHs aggregate data and send it to BS. Therefore, energy consumption is decreased, as shown in Figure 5.

**Figure 6:**
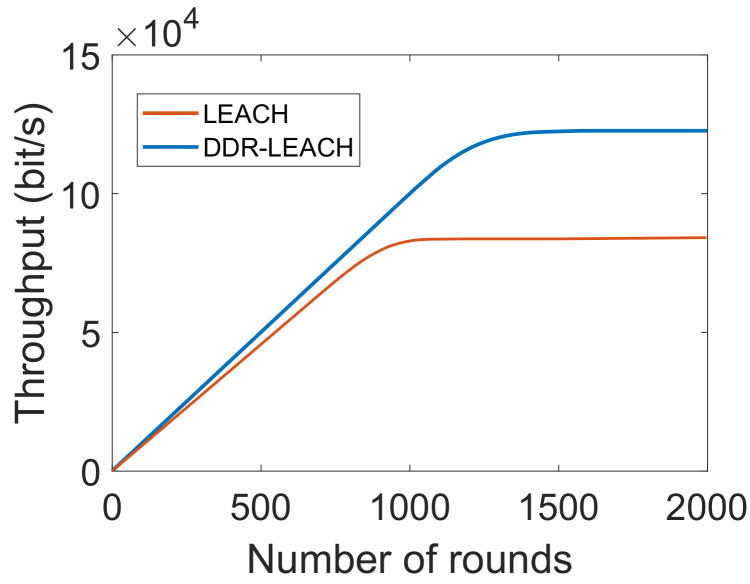
Network throughput.

Figure 6 depicts that the network throughput of both schemes is zero at the start. The reason is that no data packet is sent at initials rounds. It continues to increasing with the number of rounds because large amount of packets are sent at these rounds. In DDR-LEACH, the amount of data sent from ordinary nodes to BSs is increased gradually because all the nodes are participating in the network. It gradually decreases at 1500th round, trend becomes constant. The throughput is maximum because CHs are selected using DDR-LEACH and maximum nodes are alive to send the data packets. The data packets are increased with the decrease in the number of rounds. Whereas, in LEACH, the nodes are randomly selected and if the CHs are selected that are away from BSs, then nodes have to utilize more energy. Additionally, in DDR-LEACH, the selection of CHs is based on three parameters. Whereas, in LEACH, the randomly selected CHs die because these parameters are not considered in the CHs’ selection process. Therefore, nodes inefficiently perform operations in the network and minimum data packets are sent from source to destination.

**Figure 7:**
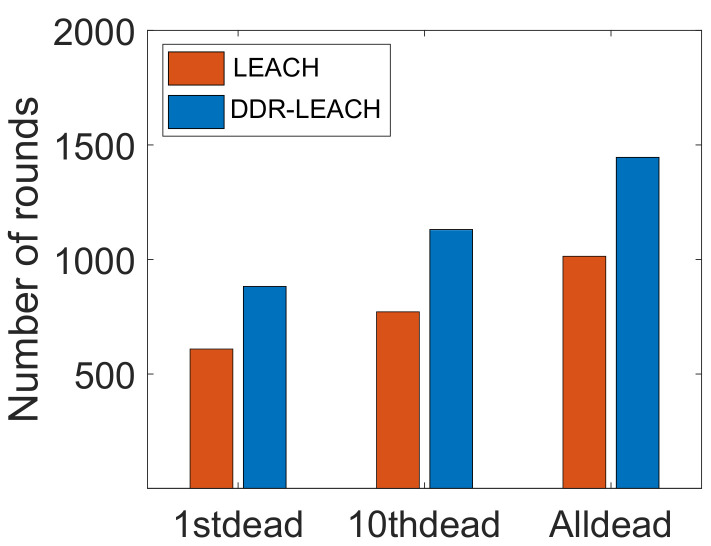
Network lifetime.

Figure 7 depicts the network lifetime of nodes. The DDR-LEACH is compared with LEACH in terms of network lifetime. In LEACH, the nodes die at an early stage like the first node dies at 600th round. Although, the 10th node dies at 750th round. All the nodes in LEACH die at 1000th round. The nodes die early in the LEACH because these nodes consume more energy and there is no mechanism for efficient CH selection. Therefore, random selection of CHs makes the network inefficient and it affects the networks’ performance. In the comparison of LEACH, the total number of rounds is 2000 in which the network nodes operate. The energy of the first node depletes at an early stage. Whereas, the 10th node dies at 1150th round. Although, all nodes die at the 1500th round. It shows that the network has a good lifetime as it operates for a large number of rounds.

**Figure 8:**
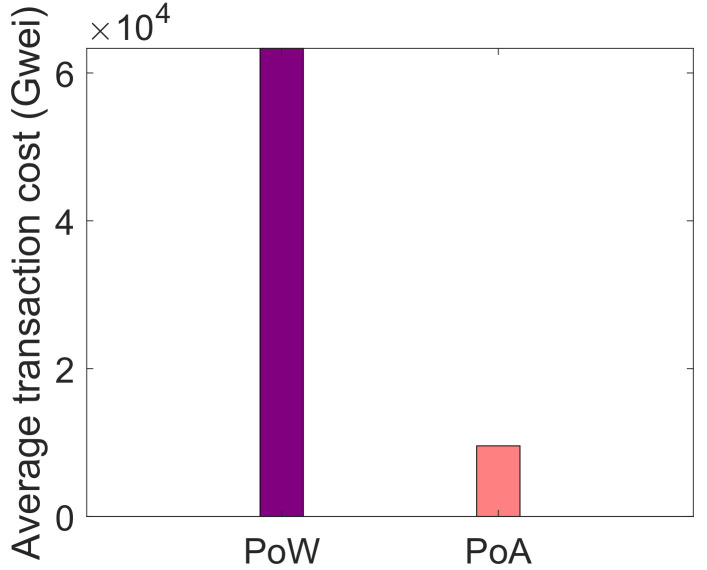
Average transaction cost for service provisioning.

**Figure 9:**
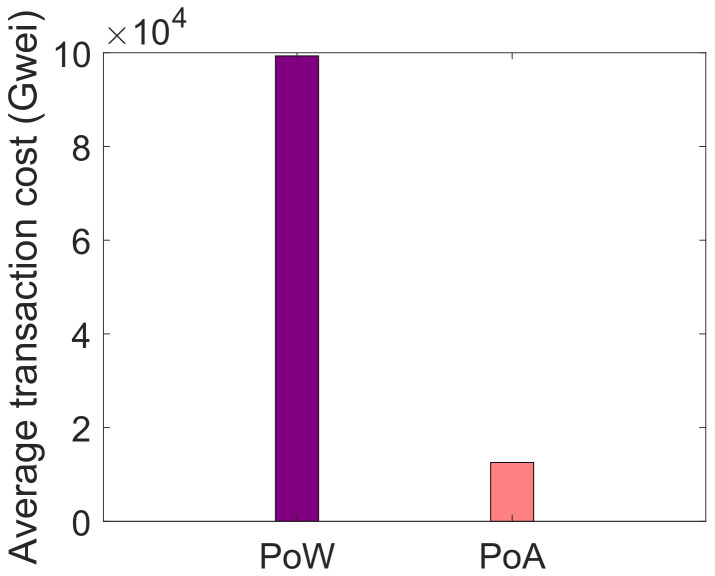
Average transaction cost for data storage in IPFS.

Figure 8 and Figure 9 illustrate the comparison of PoW and PoA consensus mechanism in service provisioning and data storage, respectively. In the proposed system model, PoA consensus mechanism is used that incurs less computational cost because in the proposed system model, private blockchain is used. Whereas, the PoW works efficiently in the public blockchain. Both consensus mechanisms are compared and their computational cost is evaluated in terms of Gwei. In the comparison of both consensus mechanisms, PoA performs better than PoW. It is because, in PoA, no mathematical puzzle is being solved by the mining nodes. In PoA, the miners are pre-selected and are responsible for validating the transactions. It is the reason that PoW incurs a large computational cost as compared to PoA. In Figure 8, when the buyers request blockchain for services, a smart contract is deployed in which PoA consensus mechanism is used. PoA, in terms of service provisioning, is compared with PoW and it is observed that PoA consumes less computational cost than PoW. On the other side, in Figure 9, when data are stored in IPFS, its average transaction cost is calculated. As discussed above, the average transaction cost of PoW is more than the average transaction cost of PoA.

**Figure 10:**
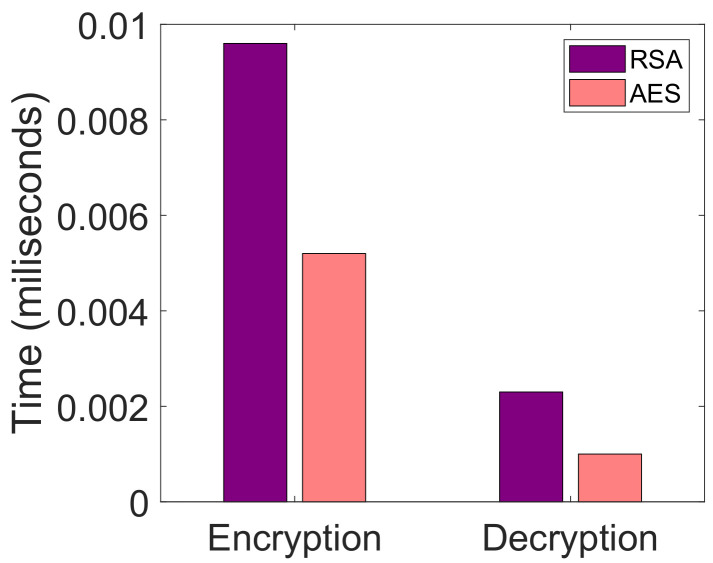
Comparison of execution time for AES and RSA.

In the proposed system model, AES 128-bit symmetric encryption and decryption scheme is used for service provisioning. Figure 10 depicts the encryption and decryption time comparison using AES 128-bit and rivest shamir adleman (RSA) schemes. AES 128-bit scheme is compared with RSA to show that AES 128-bit works efficiently in terms of time complexity. Decryption time is less than encryption times of AES, which indicates that AES performs efficiently during encryption and decryption processes. In both 256-bit and 192-bit schemes, the last rounds are asymmetric due to the absence of a mix column layer. An AES 128-bit scheme takes input data and uses a 128-bit length key $K_{Pr}$ to encrypt the plain text. The key size varies according to different key lengths in AES. Other AES schemes have different key lengths, such as 256-bit, 192-bit, etc. This paper uses AES 128-bit encryption because it takes a short time for encryption and decryption as compared to AES 192-bit and AES 256-bit [45]. In AES 256-bit scheme, there are 14 rounds while there are 12 rounds in AES 192-bit scheme to meet the encryption and decryption processes [46,47]. Moreover, the symmetric technique takes less time to encrypt the data as compared to asymmetric encryption. Additionally, for normal security purposes, AES 128-bit is enough while AES 192-bit and AES 256-bit are made to resist against the quantum computing based brute force attack, which is usually used in military based critical matters. When buyers request for the services, they are provided with the services in an encrypted form. The reason to encrypt the services is to prevent the data from unauthorized access.

## 5. Formal Security Analysis

The formal security analysis is performed to detect the malicious nodes in the network. The authentication mechanism is used specifically for the malicious nodes’ detection. The Sybil and MITM attacks are induced in the network to check the robustness of the network. Moreover, the identity information of the network nodes is stored on the blockchain and is analyzed using Oyente tool. The attacks’ analyses are given below.

*Sybil attack:* The authentication mechanism is used to make the network free from malicious nodes. In the authentication mechanism, the nodes are registered and authenticated in the network before any task is performed by them. The nodes’ information is stored in the blockchain network. A unique identity is provided to every node and its credentials are also stored in the blockchain. Therefore, this attack is not possible in this system model because nodes are mutually authenticated before performing any task.

*MITM attack:* The MITM attack is induced in the DDR-LEACH protocol, which interrupts the existing conversion in the network. To make the network free from these types of attacks, nodes’ authentication mechanism is used. Only those nodes take part in the network whose credentials are stored in the blockchain network. Before communication, these nodes have to be authenticated first. When the attacker intercepts during the registration process, the node is validated with the provided information. In the registration process, the node is provided with a unique key that is used during the authentication process. The provided key is already stored in the blockchain network. Whenever, an attacker wants to become a part of the network, it first needs to provide the exact information as the legitimate node has, which is not possible in this scenario. Therefore, attacker node is detected during the registration phase. Additionally, in the authentication phase, the attacker node needs the unique identity that is stored in the blockchain. Therefore, attacker must be verify its information using the blockchain provided key.

**Figure 11:**
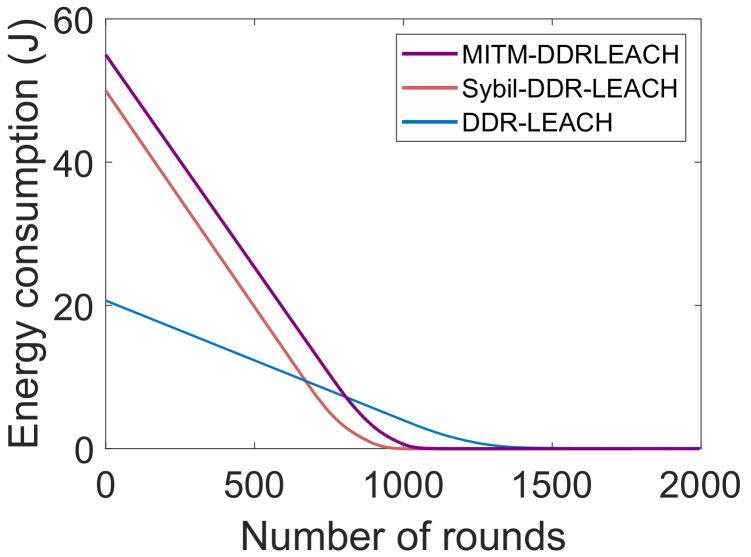
Security analysis of attacks with the proposed solution in terms of energy consumption.

Figure 11 shows that the attacker intercepts during the communication and tamper the data packets. In the MITM attack, the attacker performs the malicious activity by sending the wrong information or tampering the data packet. When the data are sent from sensor nodes to CHs and CHs to BSs, due to malicious activities, the original packet is not received at the destination. The attackers send the malicious packets again and again towards the destination. Therefore, the nodes are provided with unique key identities. Every node in the network is authenticated before it communicates with any other legitimate node. Additionally, in the Sybil attack, the attacker node makes multiple identities and manipulates the whole network. When the attacks are induced in the network, its throughput decreases because only malicious packets are sent to the destination point. After the detection of attackers through mutual registration and authentication, the network performance is improved. The throughput increases when the network is free from the attackers.

**Figure 12:**
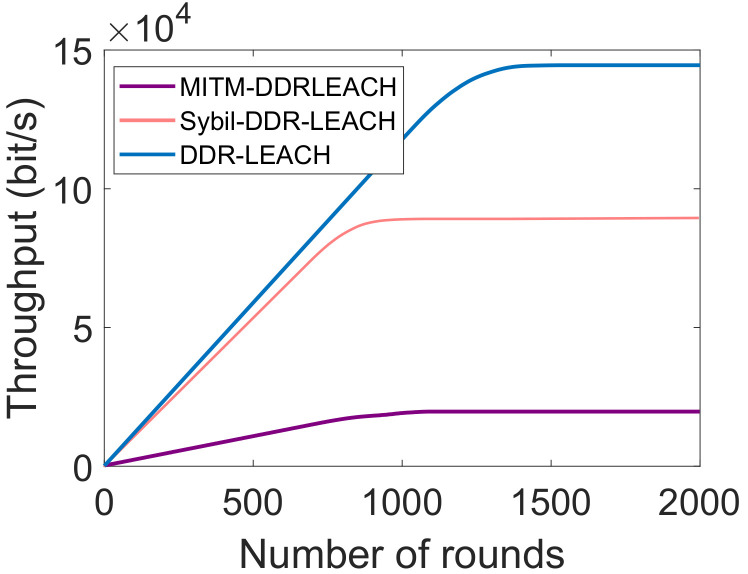
Security analysis of attacks with the proposed solution in terms of throughput.

In Figure 12, when the attackers are present in the network, the energy consumption is maximum. The attackers send the malicious packets to the destination point, due to which much energy is consumed. Whereas, after the nodes’ registration and authentication, only legitimate nodes become the of the network. Therefore, their energy consumption is minimum as compared to the energy consumed in the presence of attackers.

**Figure 13:**
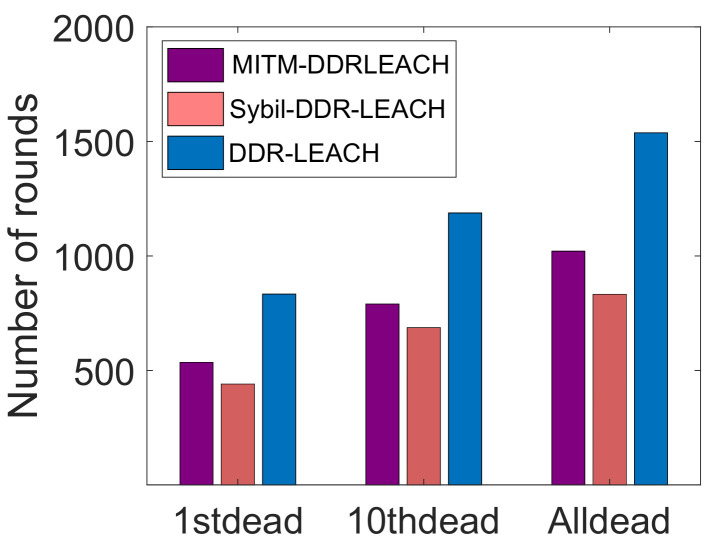
Security analysis of attacks with the proposed solution in terms of network lifetime.

Figure 13 illustrates the network lifetime in the presence of attackers and without their presence. When the attackers are present in the network, they only send the malicious data packets in the network. Additionally, they consume high energy. Therefore, the data packets sent by the legitimate nodes do not reach the destination. When the nodes’ authentication is performed, the malicious nodes are removed from the network. Therefore, the energy consumed by the legitimate nodes is less and the nodes do not die early. Whereas, in the attacker model, when the malicious nodes are present in the network, the nodes die early because their energy depletes in sending the wrong data packets to disturb the traffic.

### Smart Contract Analysis

To register and authenticate the network nodes, a smart contract is written in the solidity language. Additionally, service provisioning mechanism is used for providing the services to the buyers. The security analysis is performed on the smart contract to check its vulnerability. The tool that is used for the security analysis is Oyente. It is an open source tool that checks the possible vulnerabilities in the smart contract. The reason to perform the security analysis is the bad programming practices. Therefore, attacks are also performed such as DAO [48]. Additionally, all the business rules of our network are stored on smart contract. All sellers and buyers communicate with each other by following these business rules. Moreover, all the conditions for detecting and revoking malicious nodes in the network are also stored in the smart contract. The smart contracts provide basic infrastructure for our blockchain-based authentication and CH selection scheme. Therefore, we have provided the formal security analysis of our smart contract because it is responsible for managing every single transaction of our network. Furthermore, in blockchain based schemes, the security analysis of entire solution is performed by formal analysis of smart contact, as performed in [49,50,51]. The attacks that are possible on the smart contract are discussed below.

**Figure 14:**
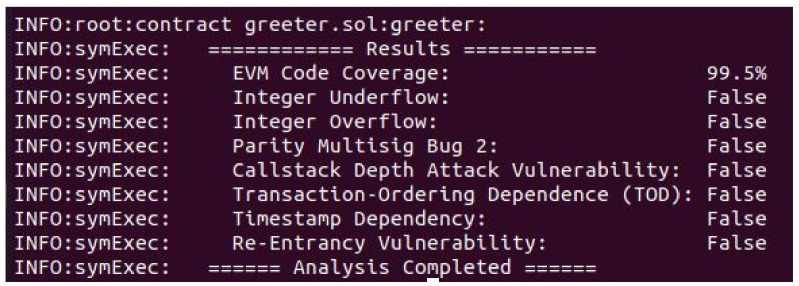
Security analysis of smart contract during registration and authentication of nodes.

Figure 14 explains the security analysis of nodes’ registration and authentication smart contract using the Oyente tool. It shows that the smart contract is resilient against the attacks that are shown in the figure. The result shows that the mentioned attacks in the figure are not possible on this smart contract. However, some attacks that are very close to vulnerabilities for the smart contract are discussed below.

*Re-entrancy attack:* When a function runs in the smart contract, the malicious node in the network calls the external function and stops the working of actual function. In our smart contract, this attack is not possible because all nodes in the network are authenticated before performing any task. The IDs of legitimate nodes are stored on the blockchain. Therefore, when nodes are not authenticated due to wrong information, then they are removed from the network.

*No double spending:* In our system model, the buyers are authenticated using the secret key that is provided to them. Therefore, no malicious node can obtain the data and perform any malicious activity in the network.

*Denial of service attack:* The denial of service attack is not possible in our system model because the buyers are authenticated by providing the secret keys for communication and they have to exchange the keys before receiving data.

*Single point of failure:* In our system model, IPFS is used for storage, which is a distributed network. Whereas, in the centralized storage system, the system is not able to give responses frequently. In our proposed system, due to distributed use IPFS, this attack is not possible. The system quickly responds to data storage and data provisioning.

## 6. Conclusions and Future Work

This paper presents a blockchain and IoST based network to minimize malicious activities and incur less computational cost. Nodes’ authorization is ensured using authentication scheme. Only authorized nodes are allowed to take part in the network and send data to CHs for further operations. CHs aggregate the data due to which their energy depletes rapidly and they die. In that case, we propose a DDR-LEACH protocol in which CHs are selected from the ordinary nodes based on their maximum degree, minimum distance from BS and maximum residual energy. CHs with low energy are replaced with nodes that satisfy the above criteria. Moreover, the aggregated data are stored in the IPFS. For long-term storage, blockchain gives incentives to IPFS. The services are provided in an encrypted form using AES 128-bit encryption scheme. The simulation results show that less computational cost is incurred during data storage and service provisioning. Moreover, low transaction cost is incurred during the registration and authentication phases. The average transaction costs incurred by PoW and PoA during data storage and service provisioning are compared and it is observed that PoA outperforms PoW. Furthermore, efficient energy consumption, network lifetime and throughput are shown in the results that are conducted by comparing LEACH with DDR-LEACH. The results show that DDR-LEACH DDR-LEACH outperforms LEACH. The encryption scheme AES 128-bit used in the proposed work shows better performance than RSA in terms of execution time. The formal security analysis is performed to check the effectiveness of smart contract against attacks. Two attacks, MITM and Sybil, are also induced in the proposed network to show the its resilience against cyber attacks. In the future work, efficient machine learning technique will be used for the malicious nodes’ detection in the network. 

## Figures and Tables

**Table 1:** Related work.

Limitations Already Addressed	Contributions Already Provided	Validations Already Done	Limitations to be Addressed
Data security and data privacy, huge energy consumption of resources, low computation power of resources, nodes’ authentication, trust issue [13]	Decentralized blockchain, public key infrastructure for resolving trust issue, nodes’ authentication	Reputation level	Authors will evaluate all parts of authentication process
Malicious nodes’ detection, malicious nodes’ traceability [17]	Trust mechanism, consortium blockchain	Sensor nodes’ data input and output parameters, credit of sensors	PoW uses more computational power, no reward for sink nodes
Mobile nodes’ management, data protection [19]	Uncertainty principle, Voronoi cell architecture, Blockchain	Network lifetime, energy consumption, average end-to-end delay, packet delivery ratio	No storage mechanism, no registration and authentication
No encryption and certificate scheme, nodes’ authentication [20]	Blockchain, SHA 64-bit algorithm, crypto based authentication	Security analysis	Node battery issue, storage issue
Node authentication, security issue, centralized system [21]	Hybrid structure is performed, Keccak hash function, consortium blockchain	Security analysis	PoA should be used for each validation and private blockchain
Data latency, limited data bandwidth, data security [22]	Blockchain based SDN, PoA, Argan2	Transactions per second, average time per block, latency	PoW consumes more computational power
Trust issue, central authority, gray hole and black hole attacks in an untrusted network [25]	Blockchain based routing protocol for route establishment, reward to minimize selfish behavior	Route overhead, packet delivery ratio, gray hole attack, black hole attack	Proposed solution must be used for ad-hoc network
Data privacy, untrusted nodes [26]	Decentralized blockchain based authentication scheme	Energy consumption	N/A
PoW takes more computational power [27]	Blockchain incentive mechanism, SHA-256	Pairing is performed by the hyper elliptic curve for the finite field	Proof of retrievability is used for recovering data in less time
Computationally extensive PoW-based mining [28]	Computation offloading mechanism	Net revenue of computing, average delay	Try different consensus mechanisms
Single point of failure, data storage [29]	Block offloading filter, blockchain	Comparison of PoW and synergistic multiple proof	N/A
Data storage, slow information validation in blockchain [30]	Blockchain distributed ledger, Tangle based technology to minimize computational time	Age of information vs sampling interval, processing power vs sampling interval	N/A
Data transparency [31]	Decentralized blockchain	Probability of attack detection by system, falsification attack, authentication delay and probabilistic scenario	No routing path is defined in order to reach the manager
No data privacy protection [32]	Blockchain-based privacy protection mechanism, double SHA-256	Data about noise	Scaled experimental data will be collected for better and complete judgment, algorithm will be improved for better result
Data privacy and data security [33]	Information centric network, public key cryptographic scheme, two-tier structure, SHA-1	Processing time, response time	Scheme should be used as practical implementation
Localization, network security [34]	Decentralized blockchain-based trust management model	Energy consumption, localization error, average error ratio	Dynamic behavior of nodes
Nonrepudiation [35]	Nonrepudiation mechanism, homomorphic hash function	Transaction latency, throughput, gas consumption	No user authentication, double spending
Malicious nodes’ detection, data security [36]	Trust aware routing algorithm	Time complexity, throughput	No authentication mechanism

**Table 2:** Mapping between limitations, solutions, and validations.

Limitations Identified	Solutions Proposed	Validations Done
L1. Nodes are not authenticated [19]. L2. No mechanism for malicious nodes’ detection [19]	S1. Authentication mechanism	V1. Message size, as shown in Figure 3 V2. Transaction cost, as depicted in Figure 4
L3. Inefficient energy consumption [21]	S2. CHs’ selection considering nodes’ residual energy, minimum distance from BS and degree	V3. Energy consumption, as depicted in Figure 5 V4. Throughput, as shown in Figure 6 V5. Network lifetime, as shown in Figure 7
L4. High computational cost [17,22]	S3. PoA	V6. Average transaction cost, as shown in Figure 8
L5. Costly data storage [19]	S4. IPFS	V7. Average transaction cost, as shown in Figure 9 V8. Encryption time, as depicted in Figure 10

**Table 3:** Simulation parameters.

Parameters	Value of Parameters
Sensing area	100 × 100 m^2^
Deployment	Random
Total nodes	100
CHs	4
BSs	2
Network interface	Wireless

## Data Availability

This study did not report any data.

## Abbreviations

**Notation**	**Description**
AES	Advance encryption standard
BSs	Base stations
CHs	Cluster heads
DDR	Distance degree and residual energy
IoT	Internet of things
IoST	Internet of sensor things
IPFS	Interplanetary file system
LEACH	Low energy adaptive clustering hierarchy
MITM	Man in the middle
PoA	Proof of authority
PoW	Proof of work
RSA	Rivest shamir adleman
SHA	Secure hashing algorithm
SPOF	Single point of failure
WSNs	Wireless sensors networks
$K_{Pr}$	Private key
